# Supplementary material for: Sex- and caste-specific developmental responses to juvenile hormone in an ant with maternal caste determination
Source: J Exp Biol. 2024 Jun 24;227(12):jeb247396. doi: 10.1242/jeb.247396 (PMC11418025; doi:10.1242/jeb.247396)
Supplement: Supplementary information [file jexbio-227-247396-s1.pdf]

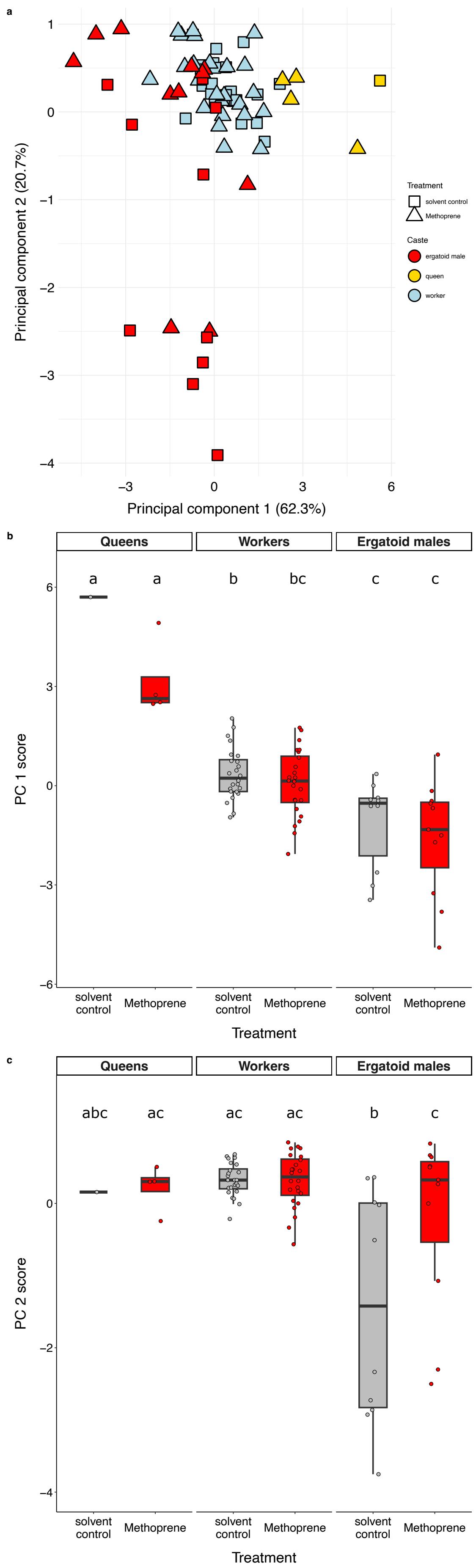

Journal of Experimental Biology • Supplementary information

Fig. S1. Body size of adult queens, workers and males after hormone treatment of early-stage embryos

a) Principal component analysis separates individuals by overall body size on PC1 and by petiole width on PC2.
b) PC 1 scores plotted by caste and treatment show that queens are the largest caste, and workers are larger than males. Overall size is not affected by hormone treatment in any of the three groups. See supplement table S4 for Tukey-corrected pairwise p-values.
c) PC 2 scores plotted by caste and treatment show a significant difference between treatment in males but not females.

Journal of Experimental Biology • Supplementary information

Table S1. Survival and caste/morph ratios following treatment of larvae with 2 µl of 1 mg/ml hormone solution

Developmental stage	Caste	Treatment	n	Survival	Queens	Workers	Ergatoid Males	Winged Males	Proportion winged individuals	Fisher's test within stages & between treatments (p<0.05)
1st instar larva	unknown	handling control	123	34.1% (42/123)	20	22	0	0	47.6% (20/42)	ab
		solvent control (70% ethanol)	224	9.8% (22/224)	9	13	0	0	40.9% (9/22)	ab
		solvent control (acetone)	66	31.8% (21/66)	10	11	0	0	47.6% (10/21)	ab
		JH III (in 70% ethanol)	129	11.6% (15/129)	3	12	0	0	20% (3/15)	a
		Methoprene (in 70% ethanol)	157	11.4% (18/157)	12	5	0	1	72.2% (13/18)	b
2nd instar larva	unknown	handling control	113	49.6% (56/113)	10	43	3	0	17.9% (10/56)	a
		solvent control (70% ethanol)	102	47.1% (48/102)	13	33	2	0	27.1% (13/48)	a
		solvent control (acetone)	107	46.7% (50/107)	1	44	5	0	2% (1/50)	b
		JH III (in 70% ethanol)	36	50% (18/36)	4	14	0	0	22.2% (4/18)	a
		Methoprene (in 70% ethanol)	95	51.6% (49/95)	9	39	1	0	18.4% (9/36)	a
3rd instar larva	unknown	handling control	30	83.3% (25/30)	6	18	1	0	24% (6/25)	a
		solvent control (70% ethanol)	21	90.5% (19/21)	4	15	0	0	21.1% (4/19)	a
		solvent control (acetone)	23	82.6% (19/23)	4	15	0	0	21.1% (4/19)	a
		JH III (in 70% ethanol)	24	75% (18/24)	4	11	1	2	33.3% (6/18)	a
		Methoprene (in 70% ethanol)	40	40% (16/40)	7	9	0	0	43.8% (7/16)	a
2nd instar larvae	queen	solvent control (70% ethanol)	11	81.1% (9/11)	9	0	0	0	100% (9/9)	a
		solvent control (acetone)	15	73.3% (11/15)	11	0	0	0	100% (11/11)	a
		JH III (in 70% ethanol)	15	66.7% (10/15)	10	0	0	0	100% (10/10)	a
		Methoprene (in 70% ethanol)	10	70% (7/10)	7	0	0	0	100% (7/7)	a
	worker	solvent control (70% ethanol)	15	46.7% (7/15)	0	7	0	0	0% (7/7)	a
		solvent control (acetone)	20	60% (12/20)	0	11	1	0	0% (12/12)	a
		JH III (in 70% ethanol)	15	60% (9/15)	0	9	0	0	0% (9/9)	a
		Methoprene (in 70% ethanol)	33	72.7% (24/33)	0	21	3	0	0% (24/24)	a

Table S2. Tukey-corrected pairwise p-values comparing body sizes of third instar larvae emerging from treatments of second instar larvae

Morphometric measurements were subjected to a principal component analysis followed by a linear regression on principal component 1 scores. (QU=queen, WO=worker, Ctrl=handling control, EtOH=solvent control, JH=juvenile hormone III, Metho=Methoprene)

contrast	estimate	SE	df	t.ratio	p.value
QU Ctrl - WO Ctrl	2.0591	0.2856	242	7.2111	<0.001
QU Ctrl - QU EtOH	0.3542	0.2928	242	1.2097	0.9285
QU Ctrl - WO EtOH	1.6589	0.2856	242	5.8095	<0.001
QU Ctrl - QU JH	0.3112	0.2794	242	1.1141	0.9533
QU Ctrl - WO JH	2.4156	0.2903	242	8.3218	<0.001
QU Ctrl - QU Metho	0.9806	0.2856	242	3.4341	0.0158
QU Ctrl - WO Metho	2.1010	0.2956	242	7.1082	<0.001
WO Ctrl - QU EtOH	-1.7049	0.2928	242	-5.8218	<0.001
WO Ctrl - WO EtOH	-0.4002	0.2856	242	-1.4016	0.8559
WO Ctrl - QU JH	-1.7479	0.2794	242	-6.2567	<0.001
WO Ctrl - WO JH	0.3564	0.2903	242	1.2280	0.9229
WO Ctrl - QU Metho	-1.0785	0.2856	242	-3.7770	0.0049
WO Ctrl - WO Metho	0.0419	0.2956	242	0.1417	1.0000
QU EtOH - WO EtOH	1.3047	0.2928	242	4.4552	<0.001
QU EtOH - QU JH	-0.0430	0.2868	242	-0.1500	1.0000
QU EtOH - WO JH	2.0613	0.2974	242	6.9300	<0.001
QU EtOH - QU Metho	0.6264	0.2928	242	2.1389	0.3931
QU EtOH - WO Metho	1.7468	0.3026	242	5.7720	<0.001
WO EtOH - QU JH	-1.3477	0.2794	242	-4.8241	<0.001
WO EtOH - WO JH	0.7567	0.2903	242	2.6067	0.1586
WO EtOH - QU Metho	-0.6783	0.2856	242	-2.3754	0.2582
WO EtOH - WO Metho	0.4421	0.2956	242	1.4957	0.8092
QU JH - WO JH	2.1043	0.2842	242	7.4048	<0.001
QU JH - QU Metho	0.6694	0.2794	242	2.3961	0.2480
QU JH - WO Metho	1.7898	0.2896	242	6.1801	<0.001
WO JH - QU Metho	-1.4350	0.2903	242	-4.9435	<0.001
WO JH - WO Metho	-0.3146	0.3001	242	-1.0481	0.9664
QU Metho - WO Metho	1.1204	0.2956	242	3.7906	0.0046

Table S3. Tukey-corrected pairwise p-values comparing body sizes of adults emerging from treatments of late-stage embryos and larvae

Morphometric measurements were subjected to a principal component analysis followed by linear regression of principal component 1 scores. (EM=ergatoid male, QU=queen, WO=worker, Ctrl=handling control, EtOH=solvent control, JH=juvenile hormone III, Metho=Methoprene)

contrast	estimate	SE	df	t.ratio	p.value
EM Ctrl - QU Ctrl	-6.4229528	0.51077875	747	-12.574824	<0.001
EM Ctrl - WO Ctrl	-3.6245119	0.5093709	747	-7.1156634	<0.001
EM Ctrl - EM EtOH	-0.5853907	0.60923732	747	-0.9608583	0.99838874
EM Ctrl - QU EtOH	-6.2072896	0.50984708	747	-12.174807	<0.001
EM Ctrl - WO EtOH	-3.2420991	0.51169637	747	-6.3359822	<0.001
EM Ctrl - EM JH	-0.8065053	0.65130209	747	-1.2382969	0.98571987
EM Ctrl - QU JH	-6.0671436	0.51256339	747	-11.836865	<0.001
EM Ctrl - WO JH	-3.3092305	0.51036286	747	-6.4840739	<0.001
EM Ctrl - EM Metho	-2.8591987	0.62041692	747	-4.6085118	<0.001
EM Ctrl - QU Metho	-6.2514611	0.51203097	747	-12.209147	<0.001
EM Ctrl - WO Metho	-3.8781541	0.51137678	747	-7.5837508	<0.001
QU Ctrl - WO Ctrl	2.79844092	0.15956243	747	17.5382192	<0.001
QU Ctrl - EM EtOH	5.83756211	0.37036679	747	15.7615699	<0.001
QU Ctrl - QU EtOH	0.21566319	0.16107608	747	1.33889024	0.97376094
QU Ctrl - WO EtOH	3.1808537	0.16683714	747	19.0656213	<0.001
QU Ctrl - EM JH	5.61644749	0.43611451	747	12.8783781	<0.001
QU Ctrl - QU JH	0.35580921	0.16947768	747	2.0994459	0.62337139
QU Ctrl - WO JH	3.1137223	0.16270128	747	19.1376638	<0.001
QU Ctrl - EM Metho	3.56375416	0.38848244	747	9.17352712	<0.001
QU Ctrl - QU Metho	0.17149169	0.16786055	747	1.02163185	0.99720053
QU Ctrl - WO Metho	2.54479873	0.16585435	747	15.3435752	<0.001
WO Ctrl - EM EtOH	3.03912119	0.36842278	747	8.2490045	<0.001
WO Ctrl - QU EtOH	-2.5827777	0.15655442	747	-16.497635	<0.001
WO Ctrl - WO EtOH	0.38241278	0.16247589	747	2.35365858	0.43935254
WO Ctrl - EM JH	2.81800658	0.43446478	747	6.4861565	<0.001
WO Ctrl - QU JH	-2.4426317	0.16518616	747	-14.787145	<0.001
WO Ctrl - WO JH	0.31528138	0.15822607	747	1.99260069	0.69850709
WO Ctrl - EM Metho	0.76531324	0.38662952	747	1.97944853	0.70742023
WO Ctrl - QU Metho	-2.6269492	0.1635266	747	-16.064354	<0.001
WO Ctrl - WO Metho	-0.2536422	0.16146656	747	-1.5708651	0.91909971
EM EtOH - QU EtOH	-5.6218989	0.36908086	747	-15.232161	<0.001
EM EtOH - WO EtOH	-2.6567084	0.37163128	747	-7.148775	<0.001
EM EtOH - EM JH	-0.2211146	0.54815239	747	-0.4033817	0.99999975
EM EtOH - QU JH	-5.4817529	0.37282417	747	-14.70332	<0.001
EM EtOH - WO JH	-2.7238398	0.36979303	747	-7.3658496	<0.001

EM EtOH - EM Metho	-2.273808	0.51107121	747	-4.449102	<0.001
EM EtOH - QU Metho	-5.6660704	0.37209185	747	-15.227612	<0.001
EM EtOH - WO Metho	-3.2927634	0.37119111	747	-8.8708034	<0.001
QU EtOH - WO EtOH	2.96519051	0.16396264	747	18.0845493	<0.001
QU EtOH - EM JH	5.40078431	0.43502297	747	12.4149407	<0.001
QU EtOH - QU JH	0.14014602	0.16664873	747	0.84096663	0.99953574
QU EtOH - WO JH	2.89805911	0.15975238	747	18.1409449	<0.001
QU EtOH - EM Metho	3.34809097	0.38725666	747	8.64566402	<0.001
QU EtOH - QU Metho	-0.0441715	0.16500389	747	-0.2676998	1
QU EtOH - WO Metho	2.32913554	0.16296252	747	14.2924612	<0.001
WO EtOH - EM JH	2.4355938	0.43718887	747	5.57103339	<0.001
WO EtOH - QU JH	-2.8250445	0.17222348	747	-16.403364	<0.001
WO EtOH - WO JH	-0.0671314	0.16555951	747	-0.405482	0.99999973
WO EtOH - EM Metho	0.38290046	0.38968814	747	0.98258176	0.99802612
WO EtOH - QU Metho	-3.009362	0.17063239	747	-17.636523	<0.001
WO EtOH - WO Metho	-0.636055	0.16865916	747	-3.7712447	0.0095204
EM JH - QU JH	-5.2606383	0.43820333	747	-12.005017	<0.001
EM JH - WO JH	-2.5027252	0.43562734	747	-5.7451058	<0.001
EM JH - EM Metho	-2.0526933	0.56055159	747	-3.6619169	0.01412323
EM JH - QU Metho	-5.4449558	0.43758044	747	-12.443325	<0.001
EM JH - WO Metho	-3.0716488	0.43681477	747	-7.0319251	<0.001
QU JH - WO JH	2.75791309	0.1682201	747	16.3946702	<0.001
QU JH - EM Metho	3.20794495	0.39082592	747	8.20811716	<0.001
QU JH - QU Metho	-0.1843175	0.17321508	747	-1.0640963	0.99599233
QU JH - WO Metho	2.18898952	0.17127161	747	12.7808079	<0.001
WO JH - EM Metho	0.45003186	0.38793546	747	1.16006889	0.99162384
WO JH - QU Metho	-2.9422306	0.16659077	747	-17.661426	<0.001
WO JH - WO Metho	-0.5689236	0.16456909	747	-3.4570499	0.02841675
EM Metho - QU Metho	-3.3922625	0.39012739	747	-8.6952686	<0.001
EM Metho - WO Metho	-1.0189554	0.3892684	747	-2.6176166	0.27156747
QU Metho - WO Metho	2.37330704	0.16967158	747	13.9876519	<0.001

Journal of Experimental Biology • Supplementary information

Table S4. Tukey-corrected pairwise p-values comparing body sizes of adults emerging from treatments of early-stage embryos

Morphometric measurements were subjected to a principal component analysis followed by a linear regression on principal component 1 scores. (EM=ergatoid male, QU=queen, WO=worker, Ethanol=solvent control, Methoprene=Methoprene)

contrast	estimate	SE	df	t.ratio	p.value
QU Ethanol - WO Ethanol	5.34767924	1.16591329	68	4.58668694	<0.001
QU Ethanol - EM Ethanol	6.8213453	1.19811419	68	5.69340166	<0.001
QU Ethanol - QU Methoprene	2.5353564	1.27719401	68	1.98509887	0.36149128
QU Ethanol - WO Methoprene	5.62962874	1.16591329	68	4.8285141	<0.001
QU Ethanol - EM Methoprene	7.28148463	1.19315303	68	6.10272484	<0.001
WO Ethanol - EM Ethanol	1.47366606	0.42996758	68	3.42738882	0.01276239
WO Ethanol - QU Methoprene	-2.8123228	0.61694332	68	-4.5584785	<0.001
WO Ethanol - WO Methoprene	0.2819495	0.32977008	68	0.85498813	0.95569552
WO Ethanol - EM Methoprene	1.93380539	0.4159431	68	4.64920654	<0.001
EM Ethanol - QU Methoprene	-4.2859889	0.67582755	68	-6.3418381	<0.001
EM Ethanol - WO Methoprene	-1.1917166	0.42996758	68	-2.7716429	0.07458445
EM Ethanol - EM Methoprene	0.46013933	0.49913172	68	0.92187955	0.93954718
QU Methoprene - WO Methoprene	3.09427234	0.61694332	68	5.01548883	<0.001
QU Methoprene - EM Methoprene	4.74612823	0.66699282	68	7.11571112	<0.001
WO Methoprene - EM Methoprene	1.65185589	0.4159431	68	3.97135061	0.0023295

Journal of Experimental Biology • Supplementary information
